# Susceptibility for varicella and factors associated with immunity among pregnant women in a tertiary care hospital in Sri Lanka- a cross-sectional study

**DOI:** 10.1186/s12879-019-3996-1

**Published:** 2019-04-29

**Authors:** Ishara P. Premathilake, Praveena Aluthbaduge, Channa P. Senanayake, Renuka Jayalatharachchi, Sirithilak Gamage, Jude Jayamaha

**Affiliations:** 1 Department of Microbiology, Faculty of Medicine, University of Colombo, Colombo, Sri Lanka; 2 Department of Virology, Medical Research Institute, Colombo, Sri Lanka

**Keywords:** Chicken pox, Infection, Pregnancy, Seroprevalence, Sri Lanka, Varicella

## Abstract

**Background:**

Varicella during pregnancy can lead to serious maternal and fetal consequences. Although an effective vaccine is available it is not incorporated in to the routine vaccination programs in most of the Asian countries. Objectives of the study were to determine the susceptibility to varicella and factors associated with immunity, among a group of pregnant women attending to a tertiary care hospital in Sri Lanka.

**Methods:**

A hospital based descriptive cross sectional study was carried out at De Soyza maternity Hospital, Colombo. A sample of 385 pregnant women was selected. Data were collected through an interviewer administered questionnaire; presence of varicella IgG in blood was assessed by a validated commercial ELISA (Enzyme Linked Immunosorbant Assay.

**Results:**

The sample had a mean age of 28.5 years and majority was educated beyond General Certificate of Education (GCE) Ordinary Level. We found that 34% of study population was susceptible for the infection. A past history of varicella had a 89.5% positive predictive value and 53.1% negative predictive value for varicella immunity. Varicella sero-positivity was only associated with a lower educational level and number of childhood household members more than four. There was no association of sero-positivity with age.

**Conclusion:**

This study demonstrates that a significant proportion of pregnant women of the study population are varicella-susceptible. Pre-pregnancy screening and preventive strategies including vaccination should be evaluated. History of past varicella infection could be a useful screening tool to exclude patients for vaccination.

**Electronic supplementary material:**

The online version of this article (10.1186/s12879-019-3996-1) contains supplementary material, which is available to authorized users.

## Background

Varicella or chicken pox is caused by varicella zoster virus, which is a highly infectious *herpesvirus*. The primary infection causes an acute febrile illness with a generalized vesicular rash. The virus establishes latency in sensory ganglia and may be reactivated later causing a vesicular rash usually confined to a single dermatome, known as herpes zoster or shingles. Immunity to varicella after primary infection is usually lifelong but occasional re-infections had been reported however [1].

Complications of varicella are commoner in adults than in children and particularly during pregnancy. Varicella related pregnancy morbidity include viral pneumonia, encephalitis, prematurity, growth retardation, fetal varicella syndrome and neonatal varicella [2–5]. Pneumonia occurs in about 10–20% and has a higher mortality than in non-pregnant adults [2]. Varicella may cause fetal varicella syndrome in 0.5% of affected pregnancies within the first 20 weeks of gestation and the risk is highest (2%) from 13-20th week POA [2, 6, 7]. Fetal varicella syndrome is characterized by clinical features as scarring of skin, limb atrophy, cataracts and neurological abnormalities [8, 9]. Infections around the time of delivery may result in neonatal varicella, which may reach a mortality rate of 30% [8, 9].

Varicella became a notifiable disease in Sri Lanka since 2005 [10]. During the last quarter in 2016, 1198 cases had been notified to the epidemiology unit, Sri Lanka [11]; However, this may be an underestimation of the true disease burden, as all cases may not come to a health care institution. The National Varicella Surveillance reveal that most of the clinical cases occur in 20–39 age group [12], into which most of the pregnant mothers will belong.

In contrast to the temperate countries where chicken pox is a childhood disease, in tropics it occurs more commonly in adolescents and young adults [13]. This pattern of age distribution in tropics makes pregnant women more susceptible to develop primary varicella during pregnancy [13]. A recent Sri Lankan study conducted in the district of Colombo had shown that varicella seroprevalence rose from 34.5% in the 10–19 year age group to 63.1% among the 20–39 year age group and then to about 75% in those above 40 years of age [14]. A study carried out among antenatal mothers in Sri Lanka also reported an increasing seroprevalence rate with advancing age with an average rate of 77.9% [15].

Exposure to varicella during pregnancy is a common and clinically challenging scenario in Sri Lanka. The recommended post exposure prophylaxis method is with varicella specific immunoglobulin (VZIg). However, VZIG is not widely or routinely available in most state hospitals in Sri Lanka and in private sector it comes at a significant cost.

The live attenuated varicella vaccine had been available since 1984 and protects against moderate/severe disease in more than 99% after two doses [16]. This vaccine is incorporated into the national vaccination programs in some countries and significant reductions in varicella related mortality and morbidity had been detected [17]. Although available in the private sector, the vaccine is not incorporated into the National Immunization Program in Sri Lanka.

Introduction of varicella vaccine to routine immunization program has several concerns including shifting of peak age of infection towards adulthood resulting in higher mortality and morbidity and the concern of higher rates of herpes zoster through a reduction of exogenous boosting of immunity by naturally circulating VZV [18]. A recent study in United States concluded that universal childhood vaccination is not cost effective increased herpes zoster morbidity and failure to produce long term varicella immunity [19]. Due to these issues vaccination of susceptible women of childbearing age or post-partum women may be useful alternate cost effective strategies [20, 21] to prevent varicella in pregnancy.

Prior to implementation of any specific prevention strategies for the women of reproduction age group it is essential to assess the susceptibility of this population. Only a few studies had been conducted so far aiming this population in recent past, two of which detected discrepant seroprevalence rates of 62% [14] and 77.9% [15]. Additional data will be useful for developing vaccination policies for this population and to predict susceptibility.

The objective of the present study was to determine the susceptibility of pregnant mothers at a Teaching Hospital in Sri Lanka to varicella and to describe factors associated with immunity.

## Methods

### Study design and setup

A hospital based descriptive cross-sectional study was carried out among 385 pregnant mothers attending antenatal clinics of De Soyza Maternity Hospital (DMH), Colombo. DMH is one of the largest tertiary care maternity hospitals in Sri Lanka and caters patients from a wide range of socio-economic backgrounds. Study was conducted in the antenatal clinic of DMH and the Department of Microbiology, Faculty of medicine, University of Colombo from August to December 2017. Required sample size was 385, with assumed 50% prevalence (maximum sample size), Confidence interval 95%, precision 5% using Winpepi statistical software version 11.39. All antenatal clinic attendees with a confirmed pregnancy were considered eligible. A confirmed pregnancy was determined either by a positive urine HCG (Human chorionic gonadotrophin) test and/or with clinical/ultrasonic evidence of pregnancy with a period of amenorrhoea (POA) more than 4 weeks. Clinic attendees were explained about the study and informed written consent was obtained. All consenting consecutive pregnant women as per the clinic attendance register were included from each clinic until the required sample size was reached. Data were collected using a pre-tested interviewer administered questionnaire. Data included place of residence, age, POA, educational level, number of children, number of household members during the pregnant mother’s childhood (below 12 years) and varicella vaccination status/history of natural infection. A positive history of chicken pox was defined as having a generalized blistering rash with fever at least two weeks prior to sample collection. A positive vaccination history was defined as recalling having at least one dose of varicella containing vaccine at least two weeks prior to sample collection.

### Specimen processing

Presence and tire of anti-varicella IgG was determined using an IVD certified validated commercial Enzyme Linked Immuno-Sorbant Assay (ELISA) [22], as per the manufacturer’s instructions. Preliminary verification of the assay which showed 100% agreement, was done with 10 positive, 5 equivocal and 10 negative pre-tested samples, obtained from Medical Research Institute, Colombo. Samples that initially tested equivocal were then re-tested in duplicate and results of two out of the three tests was taken. For quality assurance, a 10% random-sample repeat of the entire specimen collection set was performed.

### Data analysis

Seroprevalence was calculated as the number of cases positive for varicella IgG divided by the number of examined sera. Repeatedly equivocal subjects were excluded from analysis of associated factors. The terms ‘susceptibility and ‘immunity’ are used here to represent absence or presence of varicella specific IgG. Study group was divided in to urban, rural and estate sectors according to the definitions by the Department of census and statistics, Sri Lanka.

Statistical analysis was done using SPSS version 21.0. (SPSS, Chicago, Illinois USA). For qualitative variables either Pearson chi square test or Fisher’s exact test was used and independent sample t-test was used for continuous variables. Level of significance (*P*) was set at 0 .05 using 2-tailed test of significance.

## Results

### Population characteristics

Mean age was 28.5 years (SD 5.5) with an age range of 17 to 43 years. Majority (78.4%, *n* = 302) was educated beyond GCE Ordinary Level. Of all participants, 46.8% (180/385) and 53.2% (205/385) were from rural and urban sector respectively, none from estate sector. Varicella immunity was present in 254 (66.0%) and 129 (33.5%) were susceptible for the infection. Two (0.5%) had equivocal results.

### Factors associated with immunity

A past history of natural chicken pox infection/vaccination was given by 171 (44.3%) participants and 209 (54.4%) denied a past infection/vaccination; Three could not reliably recall such a history/vaccination and were excluded from further calculations. Of the participants who gave a positive history/vaccination, 89.5% (153/171) were seropositive and among those who denied, 46.9% (98/209) were seropositive (Table 1). A positive history/vaccination was significantly associated with sero-positivity (*p* = 0.000). Sensitivity of a positive history/vaccination was 60.9% (153/251) and specificity was 86.0% (111/129). Positive predictive value of a positive history/vaccination was 89.5% (153/171) and negative predictive value was 53.1% (111/209).

**Table 1:** Association between history of chicken pox/varicella vaccination and varicella sero-status among pregnant women attending antenatal clinics of De Soyza Maternity Hospital, Sri Lanka (2016)

		Varicella IgG	
		Positive (*n* = 254)	Negative (*n* = 129)
Past history of chicken pox/vaccination	Cannot recall (*n* = 3)	3	0
	Present (*n* = 171)	153	18
	Absent (*n* = 209)	98	111

IgG- immunoglobulin G

The association between several other factors with varicella sero-status was also determined (Table 2). A significantly higher sero-positivity rate was detected among those who were educated below GCE Ordinary level than those who had a higher level of education. It was also found that participants who had more than four household members during their childhood had a higher sero-positivity rate than those who had four or less household members (*P* = 0.003). There was a significantly high rate of sero-positivity among participants having two or more children when compared to those having no children (*P* = 0.0001 compared to adjusted *P* = 0.0083).

**Table 2:** Factors associated with varicella sero-status among pregnant women attending antenatal clinics of De Soyza Maternity Hospital, Sri Lanka (2016)

	Positive	Negative	*P* value
POA (weeks)	25.5 (SD 8.3)	25.5 (SD 9.1)	Not significant^*^
Residence			*P* = 0.110^**^
Urban	142	61	
Rural	112	68	
Educational level			***P*** = 0.009^**^
Below GCE O/L	65	18	
Up to/above GCE O/L	189	111	
Number of household members			***P*** = 0.004^**^
≤ 4 members	116	79	
> 4 members	138	50	
Number of children			*P* = 0.543^**^
None	116	62	
1	89	48	
≥ 2	49	19	

^*^Independent sample t test

^**^Chi square test

*SD* Standard Deviation.

*POA* Period of Amenorrhoea.

*GCE O/L* General Certificate of Education (Ordinary Level).

**Fig. 1:**
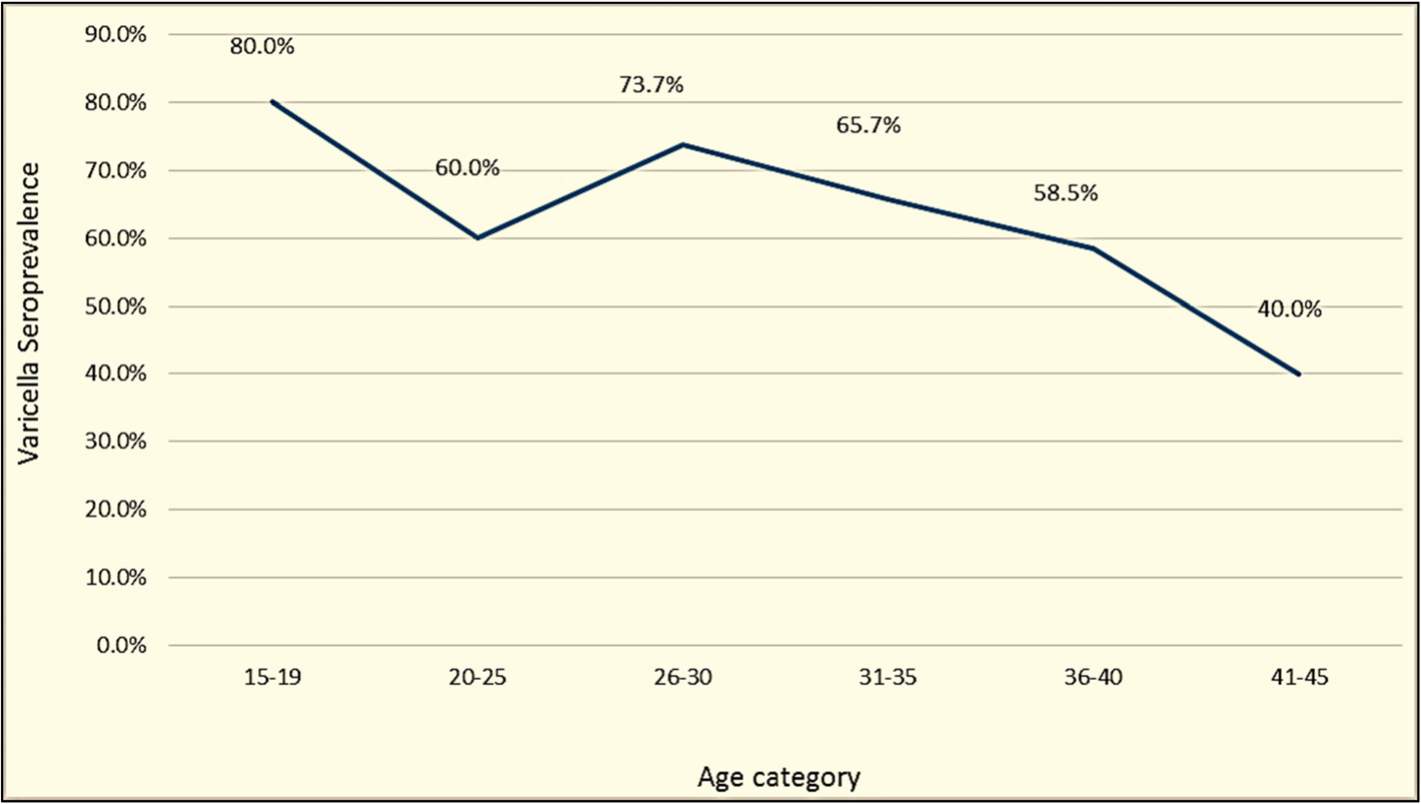
Varicella seroprevalence among different age categories of pregnant women attending antenatal clinics of De Soyza Maternity Hospital, Sri Lanka; in 2016

Participants were categorized in to seven age categories and the seroprevalence for each category was calculated (Fig. 1 and Table 3).

**Table 3:** Varicella seroprevalence according to age, among pregnant women attending antenatal clinics of De Soyza Maternity Hospital, Sri Lanka (2016)

Age category (years)	Sero-status		Total
	Negative	Positive	
15–19	4 (20.0%)	16 (80.0%)	20 (100.0%)
20–25	40 (40.0%)	60 (60.0%)	100 (100.0%)
26–30	31 (26.3%)	87 (73.7%)	118 (100.0%)
31–35	34 (34.3%)	65 (65.7%)	99 (100.0%)
36–40	17 (41.5%)	24 (58.5%)	41 (100.0%)
41–45	3 (60.0%)	2 (40.0%)	5 (100.0%)
Total	129	254	383

No significant associations between age and sero-positivity were detected. POA and sector of residence also had no significant association with the sero-positivity.

## Discussion

This study was conducted among pregnant women attending antenatal clinics of a tertiary care hospital in Colombo, Sri Lanka which serves pregnant women of different residential areas and socioeconomic backgrounds. Pregnant women were considered as the most suitable group to assess immunity to rubella in women of childbearing age (15 to 45 years) due to the ease of recruitment and as they are at risk of infection [23], which is also true for varicella.

We detected a 66% overall seroprevalence for the given population, with 34% being susceptible (non-immune). This is comparable to the 62% rate detected in the study done among women of childbearing age in Colombo district [14]. However, the study conducted among pregnant women in Peradeniya, Sri Lanka detected a slightly higher seroprevalence of 77.9% [15]. This could be due to differences in climatic factors of the two areas, where Peradeniya is situated in the central province of the country, in a cooler climatic zone and Colombo is situated in the western province in a warmer area. A Thai study described different varicella seroprevalences in four distinct climatic regions in Thailand and detected a lower seroprevalence in warmer areas [24]. Although Sri Lanka is a small country, there may be differences in seroprevalence rates across different climatic zones. However, all above Sri Lankan studies conducted among women of reproductive age group have detected seroprevalence rates generally much lower than most of the industrialized countries [25–27], which report rates above 90%. It is also somewhat lower than some of the other Asian countries as Singapore (above 84.0%) [28] and India (88.1–91.1%) [29]. Thus it is obvious that a significant proportion of Sri Lankan pregnant women are susceptible for primary varicella infection during pregnancy compared to women in most other countries.

Several developed countries have introduced varicella vaccination policies targeting different populations [17, 26]. The Centers for Disease Control and prevention recommends universal screening and vaccination of susceptible women in reproductive age group [20]. Universal vaccination against varicella for children at 18 months of age was commenced in Australia in 2005. It had been shown that following successful implementation of the campaign, there is a significant reduction of neonatal varicella rates and apparent reduction in congenital varicella rates [17]. However, implementation of a vaccination program requires identification of target populations and strategies should be individualized for each country. Pinot et al. [21] had used a stratified cohort model to assess the potential cost effectiveness of different screening strategies in UK (United Kingdom) born and Bangladesh born primi mothers. They have evaluated universal screening against verbal screening followed by serologic screening before vaccination and concluded that initial verbal screening would be more cost saving.

In our study, we found a significant association with between past history of a blistering rash or history of at least one dose of varicella containing vaccine and varicella sero-positivity. The positive predictive value and the negative predictive value of past history as a screening test for varicella immunity were 89.5 and 53.1% respectively. These demonstrate that history of varicella could be a useful screening tool in a vaccination program targeting women in reproductive age group in a developing country as Sri Lanka. However, as the negative predictive value of absence of past history and the sensitivity of the tool are low, persons without such history should undergo serological assessment before excluding immunity. A minority (10.5%) of participants who gave a past history of a blistering rash did not have detectable varicella IgG. Reasons for this could be other infectious diseases as enterovirus infections or non-infectious conditions causing blistering rashes other than varicella. There is also a possibility of waning antibody titres following infections during early life. These findings are comparable to the results of other Sri Lankan [15] and international studies [21, 25].

Among other factors, a significant association between varicella sero-positivity and educational level below GCE Ordinary Level was found. This association may be due to the possibility of linking both these factors to poor living conditions and overcrowding which increases the chances of acquiring chicken pox. However, several studies including A recent Sri Lankan study could not establish such relationship of varicella sero-positivity with educational or socioeconomic status [14, 26]. Sero-positivity was also associated with having more than four household members during childhood which also could be due to higher risk of varicella exposure. Similar association had been demonstrated with higher number of siblings in a Swiss study [25] and a Sri Lankan study conducted among adolescents [30].

Although rise in varicella sero-positivity with increasing age in many studies [16, 25, 26], we could not demonstrate such a trend among pregnant women in our study. This is mainly due to the fact that age range of our sample being 17–43 years and the number of participants in the extreme age categories were limited.

It was also shown in this study that the presence of older offspring did not influence the sero-status of the participant. According to these findings new infections during reproductive years seem occasional.

### Limitations

This study was conducted among pregnant mothers attending to a single tertiary care hospital. Thus, results of the study may not be generalizable to the whole country. However, being a tertiary care hospital the patient population was more heterogeneous than in any local hospital.

## Conclusions

This study demonstrates that the varicella susceptible pregnant population is quite significant in this set-up. Positive past history of varicella/vaccination was a useful predictor of immunity but absence of such history does not exclude immunity. Lower educational status and having more than four childhood household members were the only factors associated with immunity.

Further studies should be conducted to assess incidence of primary varicella infection in pregnant women and neonates. It is also necessary to evaluate possible screening and vaccination strategies for women in reproductive age group, including timing of vaccination.

## Additional file

Additional file 1: Data set for sero-prevalence of varicella immunity among pregnant mothers at DMH. This is the final data set used to generate the submitted article. (SAV 28 kb)

## Abbreviations

DMH: De Soyza Maternity Hospital
ELISA: Enzyme Linked Immunosorbant Assay
IgG: Immunoglobulin G
IVD: InVitro Diagnostics
POA: Period of Amenorrhoea
SD: Standard Deviation
SPSS: Statistical Package for the Social Sciences
UK: United Kingdom
VZIg: Varicella Zoster Immunoglobulin G
